# Mechanism of Wuyao–Ginseng Medicine Pair in the Prevention and Treatment of Diarrhea-Type Irritable Bowel Syndrome Based on Gene Expression Omnibus Chip Data

**DOI:** 10.3390/life13020339

**Published:** 2023-01-27

**Authors:** Wenjing Sun, Ruizi Qing, Zhiqiang Fan, Qin He, Jinhong Wu, Yang He, Linqi Ouyang, Zhen Chen, Guiming Deng

**Affiliations:** The First Hospital of Hunan University of Chinese Medicine, Changsha 410007, China

**Keywords:** GEO chip, network pharmacology, wuyao–ginseng, diarrhea-type irritable bowel syndrome

## Abstract

Based on a Gene Expression Omnibus (GEO) chip analysis combined with network pharmacology and molecular docking technology, in this study we explored the molecular targets and mechanism of the wuyao–ginseng medicine pair in the prevention and treatment of diarrhea-type irritable bowel syndrome (IBS-D). The Traditional Chinese Medicine Systems Pharmacology Database and Analysis Platform (TCMSP) was used to search for the chemical constituents and targets of wuyao and ginseng. The UniProt database was used to search for the target gene name. In the GEO database, IBS was searched to obtain GSE36701 and GSE14841 microarray data. We imported the intersection targets into the STRING database to construct a protein–protein interaction (PPI) network. Kyoto Encyclopedia of Genes and Genomes (KEGG) and Gene Ontology (Go) pathway analyses were performed using the Metascape database. A total of 30 active ingredients of wuyao–ginseng, 171 drug targets, 1257 IBS differentially expressed genes, and 20 drug-disease intersection genes were obtained from the GEO data. We screened the results and obtained the core active ingredients beta-sitosterol, DMPEC, Boldine, etc.; the core targets NCOA2, EGFR, VEGFA, etc.; and the key pathways P13K-Akt, MAPK, etc. The wuyao–ginseng medicine pair may be involved in inflammation-related signaling pathways, acting on disease targets such as NCOA2, EGFR, and VEGFA as well as pathways such as P13K-Akt and MAPK, thereby playing a key role in the prevention and treatment of IBS-D.

## 1. Introduction

IBS is a dysfunctional gastrointestinal disorder characterized by recurrent abdominal pain or discomfort and changes in bowel habits [1]. The epidemiology shows that about 5–10% of the global population suffers from IBS; the prevalence of IBS in Asia is 7% [2,3]. According to the Rome IV standards, IBS can be clinically divided into four main subtypes; namely, constipated IBS (IBS-C), diarrhea IBS (IBS-D), mixed IBS (IBS-M), and undefined IBS (IBS-U) [4]. Clinically, IBS mostly consists of constipated IBS (IBS-C) and diarrhea IBS (IBS-D). IBS-D is the most common subtype, accounting for 62.3% of cases; it causes trouble to the daily life and work of patients [5].

The TCM disease name of IBS is diagnosed as “diarrhea”, “constipation”, “abdominal pain”, etc., according to different clinical symptoms [6]. Simotang is often used in the treatment of IBS, constipation, functional dyspepsia, and other diseases [7]. Simotang originated from Jisheng Fang (Song Dynasty) and is composed of wuyao, ginseng, areca nut, and agarwood. Wuyao has a warm taste and pungent in flavour is the dry root tuber of Lindera aggregata (Sims) Kosterm. It can be a qi analgesic; warm the kidneys, dispelling cold; and regulate the liver qi as well as the upper and lower qi machinery. Numerous phytochemical studies have been conducted on L. aggregata over the last few years, reporting a great deal of sesquiterpenoids, alkaloids, sesquiterpenoid dimers, flavonoids, and phenolic acids. A few unreported novel structures, including lindenane sesquiterpene dimers and trimers, have been discovered from L. aggregata in recent years. Biological studies on the extracts and active ingredients of L. aggregata have shown multiple bioactivities such as anti-tumor, anti-inflammation, analgesia, hepatoprotection, anti-oxidation, hypolipidemia, and hypoglycemia.

Ginseng, with its bitter-sweet taste, is the dry root and rhizome of *Panax ginseng* C.A.M. Pey and can stimulate vitality without damaging positivity or the spleen and lung. Research has shown that Panax ginseng has the effects of invigorating vitality, invigorating the spleen, benefiting the lungs, and soothing the mind and eyesight. The active ingredients it contains (such as saponins, polysaccharides, volatile oils, amino acids, polyacetylenes, and various other trace elements) are also important to exert its medicinal effects.

These two medicines, one supplementing and one dispersing, can precisely target the pathogenic mechanism of the IBS intestinal barrier that causes spleen deficiencies and dampness, correcting deficiencies and evil excesses. They have the synergistic effect of supplementing, dispersing, and strengthening the intestine and stopping diarrhea [8,9]. In this study, we applied the method of a GEO chip combined with network pharmacology and molecular docking technology to elaborate on the material basis and mechanism of the wuyao–ginseng pair in the prevention and treatment of IBS-D, and to lay the theoretical basis for their clinical prescription compatibility.

## 2. Materials and Methods

### 2.1. Databases and Software 

The TCMSP Chinese Medicine Pharmacology (http://tcmspw.com), UniProt protein (https://beta.uniprot.org), Gene Expression Omnibus (GEO) (https://www.ncbi.nlm.nih.gov/geo/), STRING (https://cn.string-db.org), and Metascape (https://metascape.org) databases were used. GraphPad Prism 8 (https://www.graphpad.com), Ticket letter (http://www.bioinformatics.com.cn), and Ctyoscape 3.8.2 (https://cytoscape.org) along with the Ctyoscape 3.8.2 software cytoNCA plug-in were also used.

### 2.2. Screening of the Active Ingredients of Wuyao–Ginseng 

The ingredients of wuyao–ginseng were searched for in the TCMSP database. The parameters oral bioavailability (OB) value (%) ≥ 30 and druglikeness (DL) value ≥ 0.18 were set to screen out the eligible active ingredients contained in wuyao–ginseng, respectively. The components without corresponding targets were then screened out to obtain the relevant active ingredients [8,9]. 

### 2.3. Target Screening of Wuyao–Ginseng 

The reviewed active components of wuyao–ginseng were imported into the UniProt protein database. Homo Sapiens was selected as a species, affirmed under the “reviewed” conditions, and the drug targets were converted into a Gene Symbol. 

### 2.4. Construction of the Active Ingredient Drug Target Network of Wuyao–Ginseng 

The selected drug ingredients and drug targets were inputted into the Ctyoscape 3.8.2 software and then the active ingredient drug target network was constructed. The network was topologically analyzed with the cytoNCA plug-in. The core network between the active ingredients and the drug targets of wuyao–ginseng was then screened according to the median values of betweenness centrality (BC) and degree centrality (DC). 

### 2.5. Irritable Bowel Syndrome (IBS) GEO Chip Screening and Analysis 

Chips GSE36701 and GSE14841 were selected to search for irritable bowel syndrome in the GEO database. GSE36701 contained 40 healthy volunteers (HVS) and 53 IBS-D subjects with diarrhea as the main feature. GSE14841 consisted of 4 healthy volunteers and 5 IBS-D subjects. In the healthy volunteers and patients with IBS-D with multiple genetic differences, an absolute value |logFC| ≧ 0.5 and a *p*-value < 0.05 of genetic variations were used for the standard analysis to map the volcano and heat maps [8,9]. 

### 2.6. Construction of the Network of the Active Ingredient Disease Target for the Prevention and Treatment of IBS-D by Wuyao–Ginseng 

On the basis of the above, the drug targets and differential genes were introduced into vitalization letters. Intersection targets were generated and a Venn diagram was drawn. The network and type files were created according to the intersection genes and their active components. The files were imported into the Ctyoscape 3.8.2 software to draw the network pharmacological map. 

### 2.7. PPI Network Construction 

Multiple proteins were selected in the STRING database and the intersection genes were imported. Homo Sapiens was selected as the species and the PPI network was constructed. The tsv file of the network was then exported. After importing the tsv file into Ctyoscape 3.8.2, the cytoNCA plug-in was used to screen the core genes according to the median value of the BC and DC. The PPI network was then constructed and a visual analysis conducted. 

### 2.8. Concentration Analysis of the Drug Target Differential Genes KEGG and GO 

The Metascape database was used to carry out the KEGG and GO enrichment analysis for the differential genes of the drug target. A KEGG enrichment analysis was conducted to analyze the action pathway of the control of EBS-D by wuyao–ginseng. The top 10 items were selected according to the *p*-value for the analysis and a bubble map was drawn. The GO enrichment analysis results contained three modules: the BP (biological process); the CC (cellular component); and the MF (molecular function). The top 10 items were selected for the analysis according to their *p*-values and bar charts were drawn. 

### 2.9. Composition and Target Molecular Docking 

The core component interacted with the core target. The 3D structures of the core components and the core targets were searched for in the ZINC (http://zinc.docking.org) database and the PDB (https://www.rcsb.org/) database, respectively; these were then interconnected one by one using Auto Dock 4.2.6. The binding strength and activity of the target and active compounds were evaluated based on the Docking Score. Pymol 2.5.0 software was used for the docking analysis and drawing.

## 3. Results

### 3.1. Active Ingredients and Targets of Wuyao–Ginseng 

Through the TCMSP database, with the OB (%) ≥ 30 and DL ≥ 0.18 as the screening conditions, a total of 31 active ingredients of wuyao–ginseng was obtained. Wuyao contained 9 active ingredients and ginseng contained 22 active ingredients. The targets of the active ingredients were mapped to the certified human gene targets in the UniProt database. After the null values were removed, 30 active ingredients (Table 1) and 171 drug targets were obtained. Notably, MOL000358 was found in wuyao–ginseng.

### 3.2. Construction of the Core Network of the Active Ingredient Drug Target of Wuyao–Ginseng

**Figure 1:**
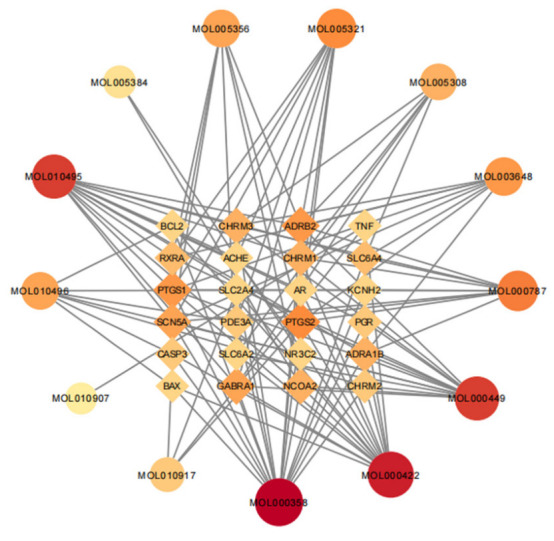
Core network of active ingredient drug targets of wuyao–ginseng.

A total of 30 active ingredients of wuyao–ginseng and their targets were imported into the Ctyoscape 3.8.2 software to construct a visualization diagram of the active ingredient drug targets of wuyao–ginseng. With the help of the cytoNCA plug-in, nodes larger than the BC and larger than the DC median value were screened (a node represented the active ingredients and drug targets of wuyao–ginseng). A new visualization network was extracted and established to construct a core network diagram of the active ingredients and drug targets of wuyao–ginseng (Figure 1). A total of 37 nodes was obtained. There were 116 edges (the edges represented the interactions between the active ingredient and the drug target). The main active ingredients were MOL000358, MOL000422, MOL000449, MOL000787, MOL010495, MOL010496, MOL005356, MOL005321, MOL005308, and MOL003648. Their corresponding targets were numerous, and included PTGS1, PTGS2, ADRB2, SCN5A, GABRA1, CHRM1, and CHRM3.

### 3.3. Analysis of GEO Differential Gene Data 

**Figure 2:**
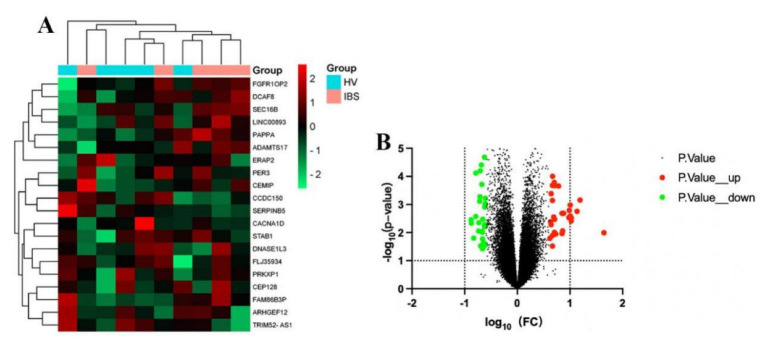
Heat map and volcano map of differential genes of HV and IBS patients with GSE36701 chip. (**A**) We selected the top 20 differentially expressed genes for the heat map (green is a low expression, black is a medium expression, and red is a high expression). (**B**) Volcano map. The X-axis was log10 (fold change). The Y-axis was -log10 (*p*-value). All genes were first set as undifferentiated genes (denoted in black) and screened according to the logFC and *p*-value. When the *p*-value was <0.05 and the logFC ≥ 0.5, it was noted as an upregulated gene (shown in red); when the *p*-value was <0.05 and the logFC ≤ −0.5, it was noted as a downregulated gene (shown in green).

**Figure 3:**
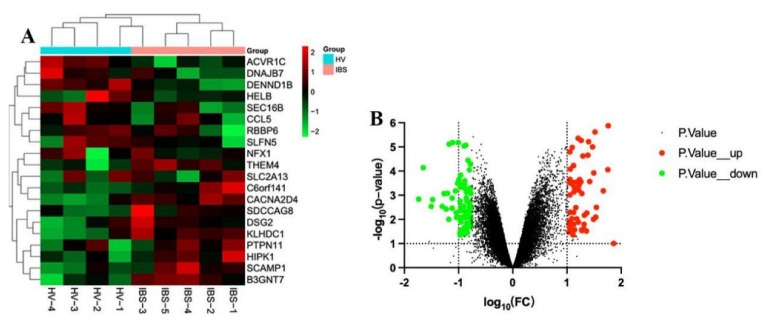
Heat map and volcano map of differential genes of HV and IBS patients with GSE14841 chip. (**A**) We selected the top 20 differentially expressed genes for the heat map (green is a low expression, black is a medium expression, and red is a high expression). (**B**) Volcano map. The X-axis was log10 (fold change). The Y-axis was -log10 (*p*-value). All genes were first set as undifferentiated genes (denoted in black) and screened according to the logFC and *p*-value. When the *p*-value was <0.05 and the logFC ≥ 0.5, it was noted as an upregulated gene (shown in red); when the *p*-value was <0.05 and the logFC ≤ −0.5, it was noted as a downregulated gene (shown in green).

The GSE36701 chip, with a total of 221 samples consisting of the selection of 40 cases of healthy volunteers (HV) and 53 cases of IBS-D subjects, was used to conduct a variance analysis using a |logFC| ≥ 0.5 and a *p*-value < 0.05 for the standard selection. A total of 160 differentially expressed genes (DEGs) was obtained. The GSE14841 chip had a sample total of 9, including 4 HV samples and 5 IBS-D subjects. A total of 1280 DEGs was obtained by screening under the same condition. According to the differential genes of each chip, the respective heat map and volcano map were drawn (Figure 2 and Figure 3). A total of 1257 DEGs was obtained by summarizing the two groups of differential genes and removing the duplicates.

### 3.4. Intersection of the Drug Targets of Wuyao–Ginseng and the Disease Targets of IBS-D

**Figure 4:**
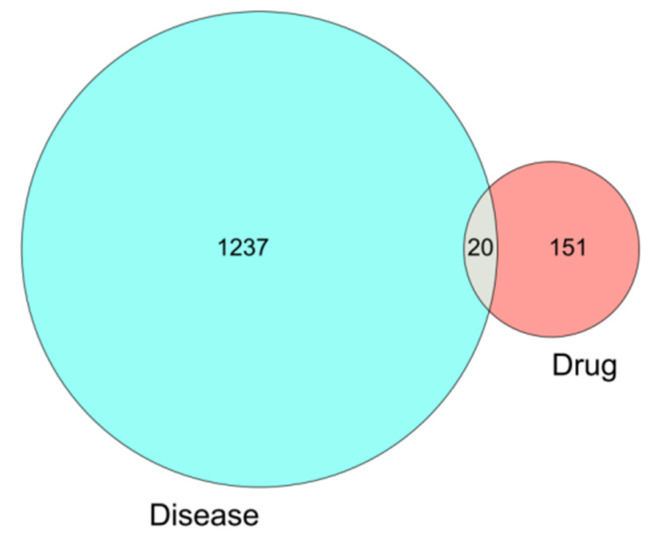
Venn diagram of the differentially expressed genes involved in IBS-D.

A total of 171 drug targets was obtained from wuyao–ginseng. A total of 1257 differentially expressed genes of IBS-D was obtained from the GSE36701 and GSE14841 chip database. From these, 20 overlapping genes were obtained (IKBKB, PPP3CA, ERBB3, NPEPPS, NCOA2, MAPK14, BCL2L1, VEGFA, EGFR, CASP9, MMP9, TOP1, ERBB2, CYP1A1, RUNX1T1, PPARA, BIRC5, INSR, DUOX2, and ADCYAP1), which were drawn as a Venn diagram (Figure 4).

### 3.5. Network Pharmacological Analysis of the Active Ingredient Disease Target of Wuyao–Ginseng

**Figure 5:**
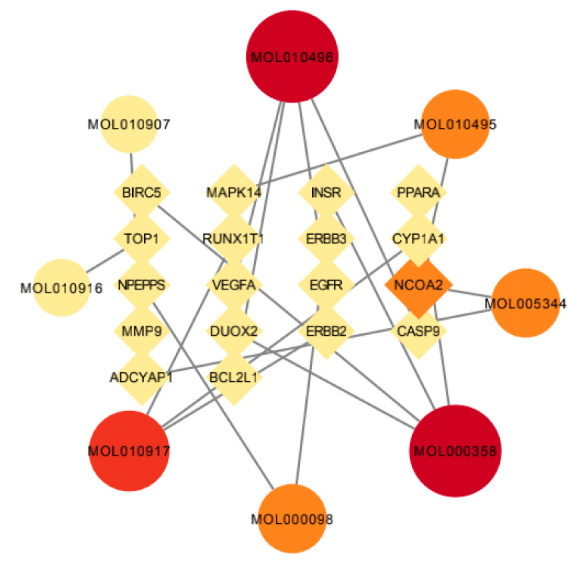
Network pharmacologic map of active ingredient disease target of wuyao–ginseng.

The active components of wuyao–ginseng and the selected intersection genes were imported into Ctyoscape 3.8.2 software to draw the network pharmacological map. Nodes larger than the BC and larger than the median value of the DC were screened out. A new visualization network was then extracted and established to construct the core network diagram of the active ingredient disease target of wuyao–ginseng, with 26 nodes and 19 edges (Figure 5). According to Table 1, the main active ingredients were beta-sitosterol, DMPEC, Boldine, 6,7-dimethoxy-2-(2-phenylethyl) chromone, quercetin, Ginsenoside, and Rh2. These results indicated that these components were the main molecules of wuyao–ginseng against IBS-D, and NCOA2 and other genes were important targets of wuyao–ginseng against IBS-D.

### 3.6. PPI Network Construction 

**Figure 6:**
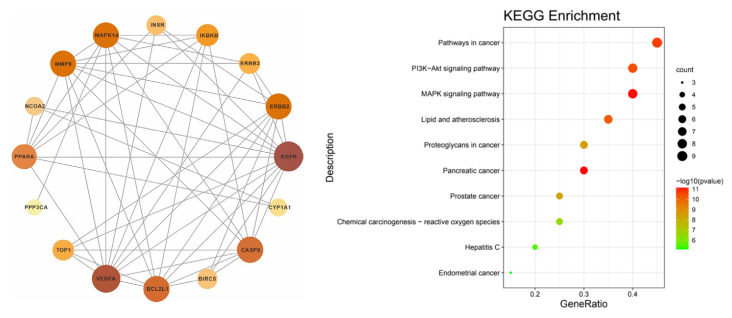
PPI network construction (**left**) and bubble map of KEGG enrichment analysis (**right**).

A total of 20 intersection genes was imported into multiple proteins in the STRING database. The species was manually selected to construct the PPI network and its tsv file was exported; this was then imported into the Ctyoscape 3.8.2 software for an analysis with the help of the cytoNCA plug-in (see left of Figure 6). The intersection gene PPI network showed that EGFR, VEGFA, BCL2L1, CASP9, MMP9, and MAPK14 were important targets. 

### 3.7. Enrichment Analysis of the KEGG Pathway 

Using the Metascape database, the intersection genes were imported and the human species was selected to obtain the enrichment analysis results of the KEGG pathway. A total of 66 channels was obtained; the first 10 channels were selected to draw the bubble map (see right of Figure 6). The results showed that pathway in cancer, the P13K-Akt signaling pathway, the MAPK signaling pathway, the lipid and atheromatocellular cirrhosis pathway, and the atherosclerosis pathway were the main pathways for the prevention and treatment of irritable bowel syndrome.

### 3.8. GO Functional Enrichment Analysis 

**Figure 7:**
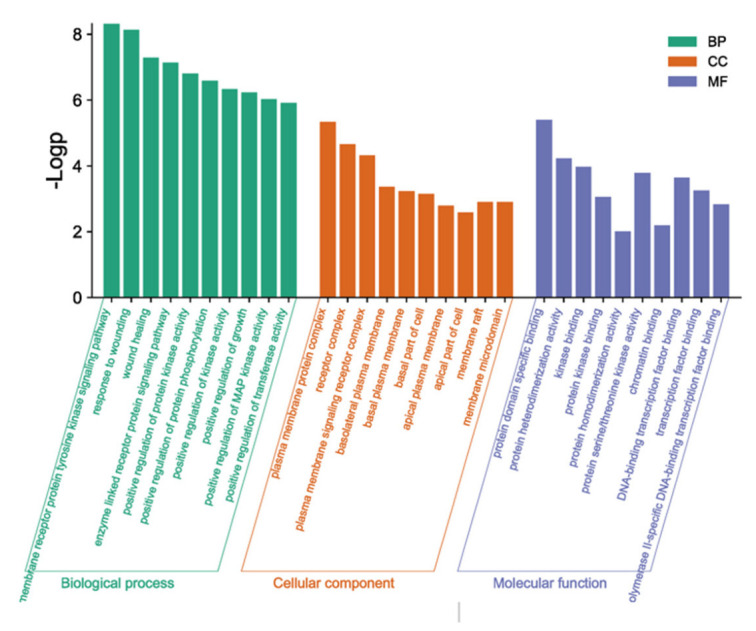
Histogram of GO enrichment analysis.

Using the Metascape database, the intersection genes were imported and the human species was selected to obtain the GO enrichment analysis results, including the biological process (BP), cell component (CC), and molecular function (MF). Through the GO enrichment analysis, 186 BF entries, 17 CC entries, and 23 MF entries were obtained. The top 10 columns were selected, respectively, according to their *p*-value to draw the enrichment analysis bar chart (Figure 7). The results showed that the BF included the transmembrane receptor protein tyrosine kinase signaling pathway and enzyme-linked receptor protein signaling pathway. The CC contained the plasma membrane protein complex and receptor complex. The MF mainly contained protein domain-specific binding and protein heterodimerization activity.

### 3.9. Verification of the Molecular Docking 

**Figure 8:**
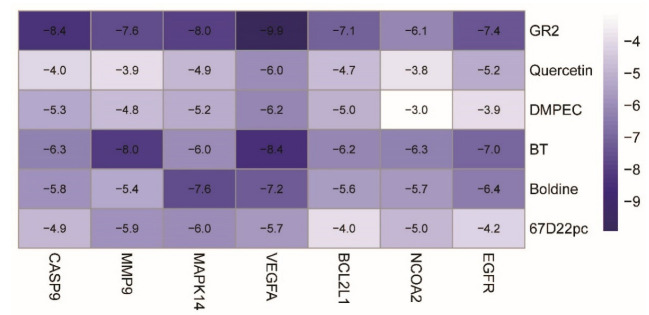
Docking heat map of PPI core target protein molecules with key compounds in wuyao–ginseng.

AutoDock software was used to rank the top 6 compounds (beta-sitosterol, DMPEC, Boldine, 6,7-dimethoxy-2-(2-phenylethyl) chromone, quercetin, and Ginsenoside Rh2), The compounds were interlinked with the 7 top core targets (NCOA2, EGFR, VEGFA, BCL2L1, CASP9, MMP9, and MAPK14) one by one, and the matching degree between the compounds and the core targets was determined from the binding energy (BE) values. In general, a BE ≤ −4.25, a BE ≤ −5.00, and a BE ≤ −7.00 Kcal/mol indicated that a compound had a certain good and strong binding energy with the target. According to the heat map analysis drawn in R4.1.2 (Figure 8), among the docking results of the 42 groups of compounds and targets, 31 groups had a good binding energy and 10 groups had a strong binding energy. 

**Figure 9:**
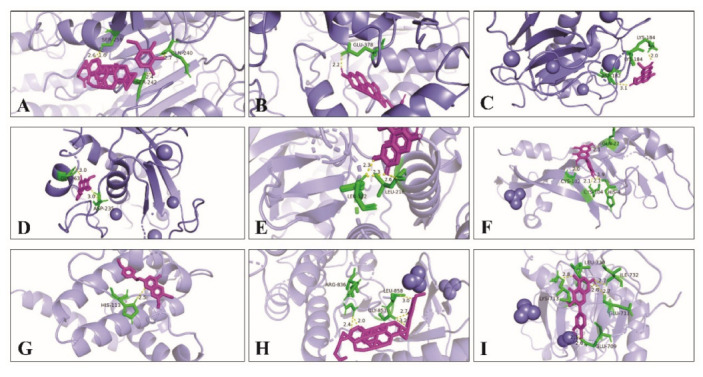
Docking model and site map of key compounds and core targets of wuyao–ginseng. (**A**) Ginsenoside Rh2 and CASP9. (**B**) Beta-sitosterol and CASP9. (**C**) Boldine and MMP9. (**D**) 6,7-Dimethoxy-2-(2-phenylethyl) chromone and MMP9. (**E**) Boldine and MAPK14. (**F**) Quercetin and VEGFA. (**G**) DMPEC and BCL2L1. (**H**) Ginsenoside Rh2 and EGFR. (**I**) Quercetin and EGFR.

At the same time, Pymol 2.5.0 software was used to analyze the docking mode of the compound and core target combination, respectively. Figure 9 shows the docking model and site map of a few key compounds and core targets of wuyao–ginseng. For example, when the compound Ginsenoside Rh2 bound to the core target CASP9, the hydrogen bond on Ginsenoside Rh2 bound to the amino acid residues of SER-242, SER-259, and GLN-240 on CASP9, respectively, with binding energies f up to −8.4 Kcal/mol. The binding activity of Ginsenoside Rh2 and CASP9 was verified.

## 4. Discussion

At present, the pathogenesis of IBS-D has not been fully elucidated. Probiotics, antibiotics, immunosuppressants, 5-HT antagonists, and fecal microbiota transplantation therapy have been widely used to treat IBS-D [10]. However, these treatments are still associated with unsatisfactory or even serious side-effects. Compared with Western medicine, traditional Chinese medicine is widely used in the prevention and treatment of functional gastrointestinal diseases due to its multi-component, multi-target, and multi-approach holistic regulation, which conforms to the characteristics of IBS-D of a multi-factor pathogenesis and multi-symptom overlap. It also has exact clinical efficacy [11]. As an important traditional Chinese medicine for the treatment of digestive diseases, wuyao is clinically used for functional gastrointestinal diseases such as chest tightness, abdominal distension, vomiting and hiccups, abdominal distensions, pain from cold and qi stagnation, and gastrointestinal neurosis such as qi stagnation [12]. The chemical components of *aconitum officinalis* mainly include volatile oils, isoquinoline alkaloids, quiterpene furans, and flavonoids [13,14], which have anti-inflammatory, analgesic, anti-rheumatic, anti-oxidation, anti-bacterial, anti-fatigue, cardiovascular protection, regulation of gastrointestinal movements, and other pharmacological effects [15,16,17]. Ginseng has functions of invigorating qi, reinvigorating the pulse, reinforcing removals, invigorating the spleen, and invigorating lungs. It is mostly used in the clinical treatment of cold bodies and limbs, deficient spleens, loose stools, shortness of breath, and fatigue [18]. Ginseng contains chemical components such as ginsenosides, ginseng polysaccharide, volatile oils (terpenoids, alcohols, fatty acids, etc.), and amino acids, which have anti-fatigue, anti-oxidation, and immunity-enhancement effects [19]. The combination of wuyao–ginseng plays an important role in regulating gastrointestinal motions and intestinal immunity in patients with IBS-D. IBS-D has a complex pathological mechanism, evidenced from a chip analysis combined with the research methods of network pharmacology and molecular docking technology [20], which are helpful to clarify the pathological mechanism of diseases and the prevention and treatment mechanism of Chinese medicine pairs. This is in line with the overall concept of Chinese medicine, which is to clarify the curative effect and reduce toxic side-effects.

In this study, a total of 171 drug targets was obtained from the TCMSP database, among which 150 targets were obtained from radix aconitum and 23 targets were obtained from radix aconitum and ginseng. A core network map of the active ingredient drug target of wuyao–ginseng was constructed to explore the active ingredients and targets of wuyao–ginseng. Subsequently, IBS were searched for in GEO database and two chip databases, GSE36701 and GSE14841, were screened. The two chip databases, GSE36701 and GSE14841, were analyzed and 1257 DEGs were obtained. These differentially expressed genes were associated with IBS-D. The intersection of 171 drug targets and 1257 DEGs yielded 20 overlapping gene targets, indicating that these genes may be the targets of the control of IBS-D by wuyao–ginseng. The active constituents and intersection genes of wuyao–ginseng were analyzed to construct an active component disease target network pharmacological map of wuyao–ginseng. The main active ingredients were beta-sitosterol, DMPEC, Boldine, 6,7-dimethoxy-2-(2-phenylethyl) chromone, quercetin, and Ginsenoside Rh2. These results suggested that these components were the main components of the control of IBS-D using wuyao–ginseng, and NCOA2 and other genes were the important targets of the control of IBS-D using wuyao–ginseng. Finally, 20 intersection genes were imported into the STRING database to construct a PPI network map. According to the intersection gene PPI network, EGFR, VEGFA, BCL2L1, CASP9, MMP9, and MAPK14 were important targets. The results of the KEGG pathway enrichment analysis showed that the cancer pathway, P13K-Akt signaling pathway, and MAPK signaling pathway were the main pathways in the prevention and treatment of IBS-D using wuyao–ginseng. The GO functional enrichment analysis showed that biological processes were related to the transmembrane receptor protein tyrosine kinase pathways and enzyme-linked receptor protein signaling pathways as well as the cell components, including the plasma membrane protein complexes. The molecular functions were related to protein domain-specific binding. AutoDock software was used to rank the top 6 compounds (beta-sitosterol, DMPEC, Boldine, 6,7-dimethoxy-2-(2-phenylethyl) chromone, quercetin, and Ginsenoside Rh2). The compounds were interlinked with 7 top core targets (NCOA2, EGFR, VEGFA, BCL2L1, CASP9, MMP9, and MAPK14) one by one and the matching degree between the compounds and the core targets was determined from the binding energy (BE) values. The results showed that the active ingredients in wuyao–ginseng were beta-sitosterol, DMPEC, Boldine, 6,7-dimethoxy-2-(2-phenylethyl) chromone, quercetin, and Ginsenoside Rh2, which may play a role in the prevention and treatment of IBS-D by improving low-grade inflammation through the NCOA2, EGFR, VEGFA, BCL2L1, CASP9, MMP9, MAPK14, PI3K/AKT, and MAPK signaling pathways.

The occurrence and development of IBS-D involve a large number of pathophysiological processes, including low-grade intestinal mucosal inflammation, increased intestinal barrier permeability, and stress [1]. Low-grade intestinal mucosal inflammation is considered to be an important pathogenic factor of IBS-D [21]. The P13K-Akt signaling pathway and MAPK signaling pathway obtained from the KEGG pathway enrichment analysis are closely related to inflammation. Studies have shown that microRNA-495 inhibits PI3K/AKT signaling via PKIB and reduces visceral sensitivity in IBS-D mice [22]. Electroacupuncture inhibited the activity of spinal astrocytes in rats with visceral hypersensitivity by inhibiting the P2Y1 reception-mediated MAPK/ERK signaling pathway, thereby improving visceral hypersensitivity in IBS-D rats [23]. These results indicate that it is feasible to prevent and control IBS-D by inhibiting the PI3K/AKT signaling pathway and MAPK signaling pathway.

Based on the above studies, it was found that the key genes were related to the PI3K/AKT signaling pathway and MAPK signaling pathway. An overexpression of NCOA2 in mouse prostate tumors led to the overactivation of PI3K/AKT and MAPK signals, thus promoting the tumor malignancy [24]. The epidermal growth factor upregulated the PD-L1 expression in OSCC cell lines through the epidermal growth factor receptor (EGFR)/PI3K/AKT pathway [25]. Ginsenoside compound K inhibited the proliferation, migration, and invasion of Eca109 cells through the VEGFA/Pi3k/Akt pathway [26]. In addition, this study found that the active ingredient of wuyao–ginseng had anti-inflammatory effects. Beta-sitosterol was shown to inhibit MAPK signaling, eliminate excessive cell proliferation and angiogenesis, and induce apoptosis, thereby preventing DEN and Fe-NTA-induced kidney cancer [27]. It is worth noting that β-sitosterol exists in both wuyao and ginseng, which may be one of the reasons that wuyao–ginseng can improve IBS-D. DMPEC and 6,7-dimethoxy-2-[2-(4’-methoxy-phenyl) ethyl] chromone exerted anti-inflammatory effects by inhibiting NF-κB activation in LPS-stimulated RAW 264.7 macrophages [28]. Boldine is a potent “natural” anti-oxidant and has a variety of health-promoting properties such as anti-inflammatory, anti-tumor, anti-diabetic, and cell protective properties [29]. These results indicated that the mechanism of the effective components of wuyao–ginseng improving IBS-D might be related to the anti-inflammatory effect.

## 5. Conclusions

In this study, by means of a gene analysis, network pharmacology, and molecular docking technology, an association analysis between drugs and diseases was conducted and the drug components, targets, pathways, and potential mechanism of action that play a key role in the prevention and treatment of IBS-D were preliminarily predicted, providing a new idea for the prevention and treatment of IBS-D by the medicine of wuyao-ginseng. 

First, a total of 171 drug targets was obtained from the TCMSP database, among which 150 targets were obtained from radix aconitum and 23 targets were obtained from radix aconitum and ginseng. A core network map of the active ingredient drug target of wuyao–ginseng was constructed to explore the active ingredients and targets of wuyao–ginseng. 

Subsequently, IBS was searched for in the GEO database and two chip databases, GSE36701 and GSE14841, were screened. The two chip databases, GSE36701 and GSE14841, were analyzed and 1257 DEGs were obtained. These differentially expressed genes were associated with IBS-D. The intersection of 171 drug targets and 1257 DEGs yielded 20 overlapping gene targets, indicating that these genes may be the targets of the control of IBS-D by wuyao–ginseng. 

Finally, 20 intersection genes were imported into the STRING database to construct a PPI network map. According to the intersection gene PPI network, EGFR, VEGFA, BCL2L1, CASP9, MMP9, and MAPK14 were important targets. The active ingredients in wuyao–ginseng were beta-sitosterol, DMPEC, Boldine, 6,7-dimethoxy-2-(2-phenylethyl) chromone, quercetin, and Ginsenoside Rh2; these may play a role in the prevention and treatment of IBS-D by improving low-grade inflammation through the NCOA2, EGFR, VEGFA, BCL2L1, CASP9, MMP9, MAPK14, PI3K/AKT, and MAPK signaling pathways. 

## Figures and Tables

**Table 1:** Active ingredients of wuyao–ginseng.

NO	MOL ID	Active Ingredients	OB (%)	DL	Drug
1	MOL010495	6,7-dimethoxy-2	31.93	0.3	Wuyao
2	MOL010496	DMPEC	32.38	0.39	Wuyao
3	MOL010907	Norboldine	40.92	0.46	Wuyao
4	MOL010913	C09495	77.09	0.25	Wuyao
5	MOL010916	Nubigenol	42.55	0.19	Wuyao
6	MOL010917	Boldine	31.18	0.51	Wuyao
7	MOL000359	Sitosterol	36.91	0.75	Wuyao
8	MOL000098	Quercetin	46.43	0.28	Wuyao
9	MOL002879	Diop	43.59	0.39	Ginseng
10	MOL000449	Stigmasterol	43.83	0.76	Ginseng
11	MOL003648	Intermit	65.83	0.54	Ginseng
12	MOL000422	Kaempferol	41.88	0.24	Ginseng
13	MOL004492	Chrysanthemaxanthin	38.72	0.58	Ginseng
14	MOL005308	Aposiopolamine	66.65	0.22	Ginseng
15	MOL005314	Celabenzine	101.88	0.49	Ginseng
16	MOL005317	Deoxyharringtonine	39.27	0.81	Ginseng
17	MOL005318	Dianthrone	40.45	0.2	Ginseng
18	MOL005320	Arachidonate	45.57	0.2	Ginseng
19	MOL005321	Frosinone A	65.9	0.34	Ginseng
20	MOL005344	Ginsenoside Rh2	36.32	0.56	Ginseng
21	MOL005348	Ginsenoside Rh4	31.11	0.78	Ginseng
22	MOL005356	Girinimbine	61.22	0.31	Ginseng
23	MOL005357	Geminis B	31.99	0.83	Ginseng
24	MOL005360	Malkangunin	57.71	0.63	Ginseng
25	MOL005376	Panaxadiol	33.09	0.79	Ginseng
26	MOL005384	Suchilactone	57.52	0.56	Ginseng
27	MOL005399	Alexandrine	36.91	0.75	Ginseng
28	MOL005401	Ginsenoside Rg5	39.56	0.79	Ginseng
29	MOL000787	Fumarine	59.26	0.83	Ginseng
30	MOL000358	Beta-sitosterol	36.91	0.75	Wuyao–Ginseng

## Data Availability

The data used to support the findings of this study are included within the article.

## References

[1] Ford A.C., Sperber A.D., Corsetti M., Camilleri M. (2020). Irritable bowel syndrome. Lancet.

[2] Black C.J., Ford A.C. (2020). Global burden of irritable bowel syndrome: Trends, predictions and risk factors. Nat. Rev. Gastroenterol. Hepatol..

[3] Sperber A.D., Bangdiwala S.I., Drossman D.A., Ghoshal U.C., Simren M., Tack J., Whitehead W.E., Dumitrascu D.L., Fang X., Fukudo S. (2021). Worldwide prevalence and burden of functional gastrointestinal disorders, results of Rome foundation global study. Gastroenterology.

[4] Drossman D.A. (2016). Functional gastrointestinal disorders: History, pathophysiology, clinical features and Rome IV. Gastroenterology.

[5] Ballou S., Mcmahon C., Lee H.N., Katon J., Shin A., Rangan V., Singh P., Nee J., Camilleri M., Lembo A. (2019). Effects of irritable bowel syndrome on daily activities vary among subtypes based on results from the IBS in America survey. Clin. Gastroenterol. Hepatol..

[6] Wu Y.N., Wang L.D., Liu J.H., Mao L.F., Du X.J., Wu X.W. (2017). Treatment of irritable bowel syndrome based on hepatic main drainage. TCM Res..

[7] Huang M.Y., Cai X.J. (2019). Research progress of clinical application of Simotang. J. Pract. Tradit. Chin. Med..

[8] Zou M.L., Huang X.Y., Chen Y.L., Ning X. (2021). Molecular mechanisms of tripterygium wilfordii in treatment of ulcerative colitis explored by GEO chip analysis combined with network pharmacology. Chin. Pharmacol. Bull..

[9] Meng X.W., Jia X.Y., Lu Z.Y., Cheng Z.L., Tan Y.N., Zhang M. (2022). Mechanism and core target analysis of Liuwei Dihuang pills in treatment of systemic Lupus erythematosus based on GEO chip and network pharmacology geo. Chin. J. Inf. TCM.

[10] Lacy B.E., Pimentel M., Brenner D.M., Chey W.D., Keefer L.A., Long M.D., Moshiree B. (2021). ACG clinical guideline: Management of irritable bowel syndrome. Am. J. Gastroenterol..

[11] Yao C.J., Li Y.L., Pu M.J., Luo L.-H., Feng P.-M. (2020). Traditional Chinese medicine for irritable bowel syndrome: A protocol for meta-analysis. Medicine.

[12] Chen H.Y., Yan J., Sun Z.G. (2015). Treating functional gastrointestinal diseases from Qi. Glob. Tradit. Chin. Med..

[13] Deng G.M., Xiang B., Xiao X.Q., Ge J.W., Chen Z., Yang L.P., Wei F. (2016). Study on chemical constituents of Lindera aggregate by GC-MS and UPLC-ESI-MS/MS. J. Chin. Med. Mater..

[14] Deng G.M., Xiang B., Xiao X.Q., Ouyang L.Q., Liu J.S., Wei F., Zhu Q., Jiang S.C. (2018). Pharmacodynamic effects of main chemical components of Lindera aggregate based on network pharmacology. Chin. Tradit. Herb. Drugs.

[15] Wang J.W., Chen X.Y., Hu P.Y., Tan M.M., Tang X.G., Huang M.C., Lou Z.H. (2016). Effects of Linderae radix extracts on a rat model of alcoholic liver injury. Exp. Ther. Med..

[16] Lu Q., Lu S., Gao X., Luo Y., Tong B., Wei Z., Lu T., Xia Y., Chou G., Wang Z. (2012). Norisoboldine, an alkaloid compound isolated from Radix Linderae, inhibits synovial angiogenesis in adjuvant-induced arthritis rats by moderating Notch1 pathway-related endothelial tip cell phenotype. Exp. Biol. Med..

[17] Gao S., Li W., Lin G., Liu G., Deng W., Zhai C., Bian C., He G., Hu Z. (2016). Norisoboldine, an alkaloid from Radix linderae, inhibits NFAT activation and attenuates 2,4-dinitrofluorobenzene-induced dermatitis in mice. Immunopharmacol. Immunotoxicol..

[18] Gao J., Lv Z.W. (2021). Research progress in chemical constituents and pharmacological action of Renshen (Ginseng). Guid. J. Tradit. Chin. Med. Pharmacol..

[19] Hou S.L., Zhang J.J., Zhang N. (2021). Fingerprints of two different varieties of panax ginseng established by HPLC. Spec. Wild Econ. Anim. Plant Res..

[20] Gui Y.R., Wang S., Dong J., Zhou T., Wang D.D., Hou W. (2022). Exploring the mechanism of Yishen Qinggan prescription to reverse endocrine therapy resistance of breast cancer based on network pharmacology. Chin. J. Inf. TCM.

[21] El-Salhy M. (2020). Possible role of intestinal stem cells in the pathophysiology of irritable bowel syndrome. World J. Gastroenterol..

[22] Fei L., Wang Y. (2020). MicroRNA-495 reduces visceral sensitivity in mice with diarrhea-predominant irritable bowel syndrome through suppression of the PI3K/AKT signaling pathway via PKIB. IUBMB Life.

[23] Zhao J., Li H., Shi C., Yang T., Xu B. (2020). Electroacupuncture inhibits the activity of astrocytes in spinal cord in rats with visceral hypersensitivity by inhibiting P2Y1 Receptor-Mediated MAPK/ERK signaling pathway. Evid.-Based Complement. Altern. Med..

[24] Qin J., Lee H.J., Wu S.P., Lin S.C., Lanz R.B., Creighton C.J., DeMayo F.J., Tsai S.Y., Tsai M.J. (2014). Androgen deprivation-induced NCoA2 promotes metastatic and castration-resistant prostate cancer. J. Clin. Investig..

[25] Kondo Y., Suzuki S., Ono S., Goto M., Miyabe S., Ogawa T., Tsuchida H., Ito H., Takahara T., Satou A. (2022). In situ PD-L1 expression in oral squamous cell carcinoma is induced by heterogeneous mechanisms among patients. Int. J. Mol. Sci..

[26] Huang J., Pan D., Liu F., Hong Y., Huang G., Huang X., Wang X., Lin Z. (2022). Ginsenoside compound K inhibits the proliferation, migration and invasion of Eca109 cell via VEGF-A/Pi3k/Akt pathway. J. Cardiothorac. Surg..

[27] Sharmila R., Sindhu G. (2017). Evaluate the antigenotoxicity and anticancer role of beta-Sitosterol by determining oxidative DNA damage and the expression of phosphorylated mitogen-activated protein kinases’, c-fos, c-jun, and endothelial growth factor receptor. Pharmacogn. Mag..

[28] Wang S.L., Tsai Y.C., Fu S.L., Cheng M.J., Chung M.I., Chen J.J. (2018). 2-(2-phenylethyl)-4H-chromen-4-one derivatives from the resinous wood of aquilaria sinensis with anti-inflammatory effects in LPS-induced macrophages. Molecules.

[29] Lau Y.S., Ling W.C., Murugan D., Mustafa M.R. (2015). Boldine ameliorates vascular oxidative stress and endothelial dysfunction: Therapeutic implication for hypertension and diabetes. J. Cardiovasc. Pharmacol..

